# Brains Don’t Predict; They Trial Actions

**DOI:** 10.3389/fpsyg.2012.00417

**Published:** 2012-10-17

**Authors:** Kevin Moore

**Affiliations:** ^1^Department of Social Science, Parks, Recreation, Tourism and Sport, Faculty of Environment, Lincoln UniversityChristchurch, New Zealand

I suggest two refinements of Clark’s (in press) account of predictive brains and situated agents. The first concerns the idea of the brain as a “predictive machine” (Clark, in press, Section 1). Simply, the brain does not *predict* how the world is but, rather, it *trials* actions that represent opportunities for adaptive ways of “going on” in the world.

The second concerns how this neuroscientific understanding relates to the action of socially situated agents (Clark, in press, Section 3.4). Briefly, *interested* social interaction employs symbolic and linguistic means to explore and trial opportunities for nuanced strategies of social action. In particular, analyses of discursive tactics (e.g., Edwards and Potter, 1992) replicate, at the interpersonal level, the “trialing” approach to achieving adaptive ends.

The main adaptive problem for cognition is not the reduction of uncertainty or to predict how the world is; it is the need to identify adaptive “ways to go on,” or act. Such a rendering of the adaptive problem for cognition usefully sidesteps notions of prediction, foresight, and other epistemological concerns. In the deepest sense, adaptive behaviors are based on “fortunate” coincidences rather than knowledge-grounded predictions. Natural selection engineers these fortunate matches.

This refinement also foregrounds emotional responses as central to so-called foresight. Emotional engagement represents a top-down “desire” about how an organism would *prefer to act* in the world. As action unfolds, those desires (preferences for action) get representationally trialed against the continuous flow of input from levels lower in the processing hierarchy.

In this way, cognition is understood in its motivational, emotional, embodied, and situated senses. This approach also emphasizes the agentic aspect of organismic behavior. Every organism has “sunk capital” (i.e., evolved neural architecture and specific embodiment) in preferred ways of acting and responding to the world. Its challenge is to use that capital to operate adaptively. Modeling the world in any disinterested manner is a luxury; quickly identifying adaptive ways to go on is a necessity.

In this conceptualization, “prediction error” is a misleading term. Information fed-forward through the neural hierarchy is not “error” (i.e., in relation to a “model”) but concerns instances of “strategic interference” (Hill and Buss, 2008).

Applying this notion of “strategic interference” more generally, perception, attention, consciousness, and the associated so-called “hierarchical predictive processing” in neural architecture are all designed to identify and respond to various obstacles (interferences or interruptions) to achieving and completing actions that have been initiated in the nervous system. The notion of “surprisal,” in its neuroscientific sense, is thus already an interested process: it is a neural event that signals likely “obstacles” to preferred action (i.e., it highlights features that require modifying actions to achieve adaptive ends).

The brain does not need to be involved in “guessing as to what is out there [in the world]” (Clark, in press, Section 4.4 para 3) through representational modeling. Instead, representations are directly related to action. As Rosenberg and Anderson (2004, no page) have argued, “what makes a given item representational is its role in providing guidance to the cognitive agent for taking actions with respect to the represented object.” In this way, a representation is a representation by virtue of it being used to represent the process of an agent performing intentional acts: “Our basic claim is that representations come into existence and derive their content from their role supporting the basic intentionality of action” (Rosenberg and Anderson, 2004).

A philosophical version of this same approach can be found in Wittgenstein’s (1953) work. In particular, it is present in his analysis of two broad ways of determining meaning: briefly, (1) meaning as something “*grasped in a flash*” (in the present context, a representation as a static model); (2) meaning as something *extended in time* (in the present context, representations of “action”). He pointed out that these two ways of determining the meaning of a word often appear to clash and so be seen as mutually exclusive. Wittgenstein’s analysis resolves the apparent clash through the intermediate notion of an “application” which has the useful conceptual qualities of being both something that can be represented “in a flash” but also requires extension over time.

A consequence of Wittgenstein’s analysis is that the search for “dual” systems in the brain that deal with the supposed dual layer of perception (representational and action-oriented perception) is not crucial. If the brain is in the business of detecting potential “applications” (of the body it coordinates) then it can have a thoroughly synthetic architecture to achieve its purpose.

In fact, the bi-directional flow of information through the hierarchy instantiates just such “applications” since it incorporates applications being “grasped in a flash” (the top-down expectation) and applications being extended over time (the bottom-up detection of instances of “strategic interference”).

This analysis also highlights “goals,” “desires” (emotions), “rewards,” and “values” as inherent in neural processing. Uses or applications are “intentional”; they incorporate ends. By contrast, as Clark (in press) notes, predictions of states of affairs are not – hence leading to a “desert landscape.” Employing the concept of an application (“pre-loaded” with intentionality) avoids the “desert landscape” while retaining its “austerity” benefits (i.e., processing and energetic requirements).

From this perspective, the insights summarized by Clark (in press) can be directly extended to agent-level cognition and experience (Clark, in press, Section 4.1). In particular, work in discursive and social constructionist psychology shows how these social and discursive “tactics” are compatible extensions of Clark’s thesis into “agent-level” phenomena.

Attempts by agents to make use of discourses in their interactions in effect trial possible social uses (applications) available in socially situated settings. As Edwards and Potter (1992) discuss, these situated acts are fundamentally “motivated” and “interested.” They achieve “discursive work” (e.g., blaming, inviting, apportioning responsibility, etc.). Discursive success relies upon skillful detection of obstacles for achieving this work. (Suggestions for an argumentative basis of reasoning and cognition provide support for this view – see Mercier and Sperber, 2011).

The above suggestions improve prospects for understanding motivated, emotionally based “foresight” as an evolutionarily grounded cognitive adaptation. They also help resolve difficulties in accounts of cognition that emphasize knowledge-based representations as guides to action.
